# Second generation lethality in RNAseH2a knockout zebrafish

**DOI:** 10.1093/nar/gkae725

**Published:** 2024-09-01

**Authors:** Ruth C Thomas, Ringaile Zaksauskaite, Norah Y Al-Kandari, Anne Cathrine Hyde, Arwa A Abugable, Sherif F El-Khamisy, Freek J van Eeden

**Affiliations:** Bateson Centre, School of Biosciences, University of Sheffield, Sheffield S10 2TN, UK; Healthy Lifespan Institute, Sheffield Institute for Neuroscience, University of Sheffield, Sheffield S10 2TN, UK; Bateson Centre, School of Biosciences, University of Sheffield, Sheffield S10 2TN, UK; Healthy Lifespan Institute, Sheffield Institute for Neuroscience, University of Sheffield, Sheffield S10 2TN, UK; Bateson Centre, School of Biosciences, University of Sheffield, Sheffield S10 2TN, UK; Healthy Lifespan Institute, Sheffield Institute for Neuroscience, University of Sheffield, Sheffield S10 2TN, UK; Bateson Centre, School of Biosciences, University of Sheffield, Sheffield S10 2TN, UK; Healthy Lifespan Institute, Sheffield Institute for Neuroscience, University of Sheffield, Sheffield S10 2TN, UK; Healthy Lifespan Institute, Sheffield Institute for Neuroscience, University of Sheffield, Sheffield S10 2TN, UK; Bateson Centre, School of Biosciences, University of Sheffield, Sheffield S10 2TN, UK; Healthy Lifespan Institute, Sheffield Institute for Neuroscience, University of Sheffield, Sheffield S10 2TN, UK; The Institute of Cancer Therapeutics, University of Bradford, BD7 1DP, UK; Bateson Centre, School of Biosciences, University of Sheffield, Sheffield S10 2TN, UK; Healthy Lifespan Institute, Sheffield Institute for Neuroscience, University of Sheffield, Sheffield S10 2TN, UK

## Abstract

Removal of ribonucleotides from DNA by RNaseH2 is essential for genome stability, and its impacted function causes the neurodegenerative disease, Aicardi Goutières Syndrome. We have created a zebrafish *rnaseh2a* mutant to model this process. Surprisingly, RNaseH2a knockouts show little phenotypic abnormality at adulthood in the first generation, unlike mouse knockout models, which are early embryonic lethal. However, the second generation offspring show reduced development, increased ribonucleotide incorporation and upregulation of key inflammatory markers, resulting in both maternal and paternal embryonic lethality. Thus, neither fathers or mothers can generate viable offspring even when crossed to wild-type partners. Despite their survival, *rnaseh2a^−/−^* adults show an accumulation of ribonucleotides in both the brain and testes that is not present in early development. Our data suggest that homozygotes possess RNaseH2 independent compensatory mechanisms that are inactive or overwhelmed by the inherited ribonucleotides in their offspring, or that zebrafish have a yet unknown tolerance mechanism. Additionally, we identify ribodysgenesis, the rapid removal of rNMPs and subsequently lethal fragmentation of DNA as responsible for maternal and paternal embryonic lethality.

## Graphical Abstract

**Graphical Abstract:**
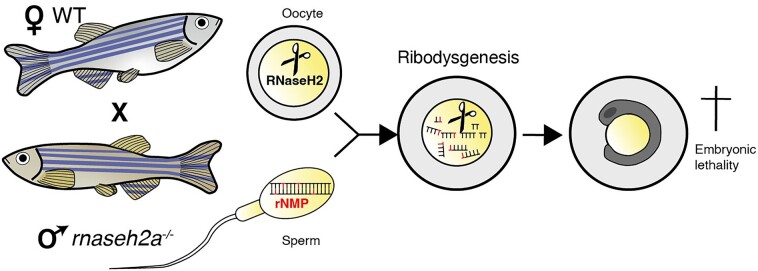

## Introduction

Maintaining genome stability is essential for the survival of organisms. The incorporation of ribonucleotide monophosphates (rNMPs) is one of the most abundant forms of DNA damage, due in part to the large ratio of ribonucleoside triphosphates (rNTPs) to deoxyribonucleoside triphosphates (dNTPS) in eukaryotic cells (1,2). The inability of DNA polymerases to completely exclude rNMPs during DNA replication are also a contributing factor (2) as well as the intentional incorporation of rNTPs during events such as non-homologous end joining (NHEJ) (3). Due to their reactive 2′ hydroxyl group, rNMPs are far more susceptible to spontaneous hydrolysis than dNMPs (2). If left unchecked, they can result in severely reduced genome stability via an increased number of DNA breaks (4–6).

Removal of rNMPs that are incorporated into the genome is preferentially performed by the Ribonuclease H (RNaseH) family of enzymes. Whereas RNaseH1 is only able to target four or more consecutive rNMPs, RNaseH2 is able to remove single ribonucleotides incorporated in dsDNA via the incision of the DNA strand at the 5′ side of the ribonucleotide creating a 3′-hydroxyl and 5′-phospho-ribonucleotide ends. The subsequent overhang is cleaved by FEN1/Exo1 and the remaining DNA gap repaired by DNA ligase (7,8). RNaseH2 is a heterotrimeric complex that includes an active subunit, A, and auxiliary subunits B and C. All three have been found to be essential for its function and mutations in each subunit have been found in patients with the severe neuro-inflammatory disease, Aicardi Goutières Syndrome (AGS) (9).

AGS is an early onset Type 1 interferonopathy that presents with an uncontrolled Type 1 interferon (IFN) response, similar to systemic lupus and congenital viral infections (10–12) Although over 50% of AGS patients have been identified with mutations in *RNASEH2A/RNASEH2B/RNASEH2C*, others have also been found with mutations in *TREX1, SAMHD1, ADAR1* and *IFIH1* (9,13–15). Recent studies have suggested that despite the increased inflammatory markers, it may be the systemic DNA damage that is the driver of the severe AGS neuropathological phenotypes such as microcephaly, seizures and intracranial calcifications (16,17). *In vivo* models to study the effect of non-functional RNaseH2 subunits have been created (18–22) along with models for other known AGS genes, such as SAMHD1 (23–25) ADAR1 (26–29) and TREX1 (30–32). So far, homozygous knockouts of all RNAseH2 subunits have not been viable past the embryonic stage in mouse.

Here, we describe a viable *Danio rerio* knockout of RNaseH2a that can survive to adulthood and reproduce, unlike previous *in vivo* models. Surprisingly, however, unlike the homozygous adults, progeny from both homozygote females and males have a severe phenotype that is not rescued by the re-introduction of RNaseH2a, resulting in a second generation, embryonic lethality.

This model provides an exciting opportunity to study the pathways compensating for the lack of RNaseH2 activity, increasing our understanding of the mutational landscape in both AGS and cancer patients, stemming from the incorporation of rNTPs.

## Materials and methods

### Zebrafish husbandry

Adult zebrafish were housed in the Bateson Centre aquarium at 28°C and a 14-h/10-h light/dark cycle. All experiments were performed in accordance with the U.K Home Office Animals (Scientific Procedures) Act 1986 under project licence PB2866EDO and PC39B259E held by F.v.E. and personal licence ID948DC95 held by R.C.T.

### Whole mount *in situ* hybridization

Probes against *rnaseh2a* (Table 1) were produced via transcription of linearized plasmid DNA (pME18S-FL3-rnaseh2a, Source Bioscience) using the Thermofisher mMessage mMachine kit, following the manufacturers instructions. 24 hpf zebrafish were fixed in 4% PFA overnight at 4°C before being dehydrated in serial incubations with 30%, 60% and 100% MeOH/PBST. Rehydration back through these dilutions was essential before continuing. Embryos were pre-hybridized in HM^+^ (50% formamide, 5× SSC, 9.2 mM citric acid, 0.1% Tween-20, 0.5 mg/ml tRNA (Invitrogen, 15401029), 0.05 mg/ml heparin) for 1 h at 70°C. The solution was removed and replaced with HM^+^ containing 500 ng of RNA probe which had been pre-heated at 70°C before being left overnight. Embryos were washed in HM^−^ (50% formamide, 5× SSC (ChemCruz, SC296419), 9.2 mM citric acid, 0.1% Tween-20, pH 6) then for 15 min at 70°C in 75% HM^−^/25% 2× SSC, 50% HM^−^/50% 2× SSC, 25% HM^−^/75% 2× SSC and 2× SSC. They were then washed for 2 × 30 min with 0.2× SSC at 70°C followed by 10-min washes at RTP with 75% 0.2× SSC/25% PBST, 50% 0.2× SSC/50% PBST, 25% 0.2× SSC/75%PBST and 100% PBST. 1 ml of blocking buffer (2% Blocking reagent (Roche, 11096176001) dissolved in 1× malate Buffer at 70°C) was added and incubated for 3 h at RTP with shaking. The blocking buffer was replaced with a 1:5000 dilution of α-DIG antibody (Roche, 11093274910) in blocking buffer and the sample was incubated overnight with gentle rocking at 4°C. Embryos were washed briefly in PBST before 4 × 30 min incubations in PBST at RTP in the dark. AP^−^ (100 mM Tris pH 9.5, 100 mM NaCl, 0.1% Tween-20) was added for 15 min, RTP in the dark before two 10 min washes with AP^+^ (100 mM Tris pH 9.5, 100 mM NaCl, 50 mM MgCl_2_, 0.1% Tween-20). Embryos were transferred to a 24-well plate and incubated with 0.5 ml Staining Solution (3.4 ul NBT and 3.5 ul BCIP in 1 ml AP^+^ buffer) (Table 2). Embryos were monitored every 30 min–1 h for stain development. Samples were washed 3 × 5 min with PBST and post fixed for 20 min in 4% PFA, RTP before successive incubations in 25%, 50% and 80% glycerol/PBS. Long-term storage at –20°C.

**Table 1.** Whole mount *in situ* hybridization primers used in this study

Target	Oligo (uppercase, gene sequence)	Direction	Template
*rnaseh2a*	taatacgactcactatagggGTTTGTGGACACTGTGGGTCatttaggtgacgctatagCTCCCGTTTTCTCCACCTCT	FR	cDNA

**Table 2.** Antibodies

Primary antibody	Reactivity	Host	Supplier	Concentration	Application
DIG-AP	-	Sheep	Roche (11093274910)	1:5000	ISH
γH2AX	Zebrafish	Rabbit	Genetex (GTX127342)	1:1000	WB
GAPDH	Zebrafish	Rabbit	Genetex (GTX100118)	1:1000	WB
pCHK1	Zebrafish	Rabbit	CST (2348)	1:1000	WB
PAR	Zebrafish	Mouse	Genetex (GTX100118)	1:1000	WB
Anti- IgG (H + L)-Alexa Fluor 488	mouse	goat	Molecular Probes (A28175)	1:2000	WB
IgG (H + L)-HRP Conjugate	mouse	goat	Bio-Rad (170–6516)	1:4000	WB
IgG (H + L)-HRP Conjugate	rabbit	goat	Bio-Rad (170–6522)	1:4000	WB

Species reactivity, host species, supplier, working concentration, and application. WB, Western blot; SB, slot blot; ISH, *in situ* hybridization; IgG, immunoglobulin G; HRP, horseradish peroxidase.

### Generation of *rnaseh2a^−/−^* zebrafish

Single cell embryos were injected with 2.4-μg Cas9 mRNA and single gRNA (sgRNA; 0.4 μg/μl), targeting exon five of *rnaseh2a* (Table 3). Cas9 mRNA was *in vitro* transcribed from 1 to 2 μg of Not1-linearized pCS2-nCas9n plasmid using the mMESSAGE mMACHINE kit (Life Technologies, AM1340), while sgRNA was transcribed with the MEGAshortscript T7 Transcription Kit (Life Technologies, AM1354), according to the manufacturer's instructions. Embryos were raised to adulthood and then outcrossed with wild-types. Subsequent embryos were sequenced to identify desired mutations. Selected founders were then outcrossed to produce a heterozygous F1 generation before being incrossed to create a stable *rnaseh2a^−/−^* zebrafish line.

**Table 3.** gRNA used in this manuscript

Target	Sequence	Source
*rnaseh2a*	AAAGCACCGACTCGGTGCCACTTTTTCAAGTTGATAACGGACTAGCCTTATTTTAACTTGCTATTTCTAGCTCTAAAACCGGTGTGGAGGTCACAGTCCCTATAGTGAGTCGTATTA	Integrated DNA Technologies (IDT)
*top1*	GAAACTGAGCCCCGCTG**CGG**TGATGTCCTCGGGTCGGATG**CGG**	Integrated DNA Technologies (IDT)
*top1l*	TGTCTTTAGTGGTGTACTCG**TGG**GAGCCTCCAGGCTTGTTCCG**TGG**	Integrated DNA Technologies (IDT)
*rnaseH1ex1 + ex2*	AACCCGGAGTGTATCAGACA**TGG**GTCCAAAGCATCCTGTTCGG**AGG**	Integrated DNA Technologies (IDT)

PAM site in bold.

### Generation of *top1/top1l* CRISPANT zebrafish, and p53 morphants

Single cell embryos were injected with 10fmol Cas9 (NEB, M0386), and 10 fmol of trans-activating CRISPR RNA (tracrRNA) and 2.5 μM of 4 g RNAs targeting desired exons (2 g RNAs in case of *rnaseh1)* (Table 3). Embryos underwent PCR analysis (Table 4) to identify a range of indels identifiable using agarose gel electrophoresis at 100 V/cm for 2 h. For p53 knockdown, ∼4.5 ng standard p53 morpholino (GCGCCATTGCTTTGCAAGAATTG; Gene-Tools) was injected, this morpholino has been verified by (33), and also by (34) on western blot.

**Table 4.** PCR primers required for genotyping of zebrafish

Target	Sequence	Direction	Source
Exon 6 of *rnaseh2a*	GTTTGTGGACACTGTGGGTC CTCCCGTTTTCTCCACCTCT	FR	Integrated DNA Technologies (IDT)
Exon 5 of *top1*	ATTGAGCCTCCAGGTCTGTTTA CACAATGCAGAAAGATGAGAGA	FR	Integrated DNA Technologies (IDT)
Exon 12 of *top1l*	TTATGGACAATCACAAAGAGCG GATAATGTCTTCGGGTCGGATA	FR	Integrated DNA Technologies (IDT)
Exon 1 of *rnaseh1*	GTACGCAGAACTCTATGCAACG ATGCAACACGCGTCAATAATAC	FR	Integrated DNA Technologies (IDT)
Exon 2 of *rnaseh1*	GGAGGAATGTAAACATCAAGTGG TGATATTACCATTTGCTCCCG	FR	Integrated DNA Technologies (IDT)

### RNaseH2a activity assay

Protein was isolated from embryos via de-yolking in ice-cold PBS before being washed twice and homogenised with a micropestle. 1–1.5 μl of lysis buffer (200 mM Hepes, 40 mM NaCl, 2 mM MgCl_2_, 0.5% Triton X-100, 1× protease inhibitor cocktail (Roche, 4693159001), 1× phosphatase inhibitor cocktail (Roche, 4906837001)) was added per embryos and incubated for 30 minutes on ice. Samples were then centrifuged at 13 300 rpm for 15 min at 4°C. The supernatant was then transferred to a fresh tube at stored at –20°C. As a positive control commercially available RNAseHII was used (NEB). Quantification of protein concentration was performed via a Bradford assay with Coomassie Plus™ Protein Assay Reagent (Thermo Scientific, 23200). The following method was modified from (35). 100/200/400 ng of protein was incubated with 0.45ul of substrate (5′-GGTAACGCCAGGGTTTTCTCrGTTCACGACGTTGTAAAACGA) in 1× NEBbuffer, 0.5 μl 100× BSA and 0.5 μ× DTT for 2 h at 37C. 13 ul of 2× Termination buffer(10mM EDTA, 80% formamide, 1% bromophenol blue) was added and incubated for 20 min at 65°C. 2 μl of 100 uM competitive DNA (5′-GGT AAC GCC AGG GTT TTC TC) was added and boiled at 95°C for 5 min. A 20% polyacrylamide 12 M urea gel was used to separate the cleavage products before visualization with the ChemiDoc MP imaging system (Bio-Rad, 1708280).

### Reverse transcription qPCR

Zebrafish embryos were homogenized in TRIzol reagent (Invitrogen) to extract RNA, according to the manufacturer's protocol. Concentration was determined using The NanoDrop (Thermo Fisher Scientific). 500 ng of RNA was reverse transcribed using the Photoscript II cDNA synthesis kit (NEB) according to manufacturer's instructions. cDNA was diluted 1:10 and 1 ul was used in combination with SYBR Master Mix (NEB) and 500nM of primers (Table 5). To produce a standard curve, 100%, 10%, 1% and 0.1% cDNA dilutions were used as templates for each primer pair. Each reaction was run in triplicate in a CFX96 Touch™ Real-Time PCR Detection System (BioRad) for 95°C for 10 min, followed by 45 cycles of 95°C for 15 s, 55°C for 15 s and 72°C for 30 s. Analysis was performed with CFX Maestro™ Analysis Software.

**Table 5.** Quantitative PCR primers

Gene	Sequence	Direction	Reference
*rps29*	TTTGCTCAAACCGTCACGGA	F	(57)
	ACTCGTTTAATCCAGCTTGACG	R	
*ISG15*	AAC TCG GTG ACG ATG CAG C	F	(58)
	TGG GCA CGT TGA AGT ACT GA	R	
*mxa*	GAC CGT CTC TGA TGT GGT TA	F	(58)
	GCA TGC TTT AGA CTC TGG CT	R	
*IL1b*	TGGACTTCGCAGCACAAAATG	F	(59)
	GTTCACTCCACGCTCTTGGATG	R	
*IFNΦ1sec*	ACG GCA GCC TGA AAT ACG TT	F	(58)
	GTC CTC CAC CTT TGA CTT GT	R	
*Il6*	GCTCATCCAGCAGGGTCCG	F	(60)
	CGACACACACTGTTTGGCCTTG	R	
*tnfa*	GCGCTTTTCTGAATCCTACG	F	(59)
	TGCCCAGTCTGTCTCCTTCT	R	
*p21*	AGGAAAAGCAGCAGAAACG	F	(61)
	TGTTGGTCTGTTTGCGCTT	R	
*top1*	ACGTACAACGCGTCCATTACGCTCGGTTGGCTCTGTTATAG	FR	Designed
*top1l*	GGATCAAGGGAGAGAAGGATTGATTTCCAGTCATCGCGGTA	FR	Designed
*Δ113p53*	CAAGACCAGGTATTCACCGGAC	Taqman Probe	(34)

### Alkaline assay

Thirty pooled embryos or individual adult tissue samples were washed in sterile water before being homogenised with a micropestle in 100 μl TNAE (Total Nucleic Acid Extraction) Buffer (100 mM Tris–HCl (pH 8.0), 100 mM EDTA, 250 mM NaCl, 1% SDS). 300 ul TNAE Buffer was added and a second round of homogenization occurred. The samples were incubated with 200 ug/ml Proteinase K for 3 h at 50°C. Phenol-Chloroform purification was performed as in and pellets re-suspended in 20 μl TE Buffer (10 mM Tris, 1 mM EDTA, pH 8). Concentrations of each sample were checked by running on a 1% agarose gel and ratios calculated to ensure equal loading. Samples were incubated with 0.3 M NaOH, or NaCl as a control, for 15 min at 55°C before being loaded onto a 1% agarose gel. Gels were run for 5 min at 65 V before 22 h at 1 V/cm. The gels were stained in SybrGold at 1:1000 overnight before being imaged by UV transillumination (Bio-Rad, 1708280). Image analysis was performed with ImageJ and graphical production with R Studio as described in Supplemental Methods.

### γH2AX immunofluorescence

24hpf embryos were dechorionated and fixed in 50% MeOH:acetone overnight followed by 5 min in 50% MeOH:PBS and PBS. Embryos are then permeabilised in 1% Triton:PBS before being incubated with Blocking Buffer (2% Blocking reagent (Roche, 11096176001) dissolved in 1× malate Buffer), 5% FCS and 1% DMSO for 3 h at 4°C. Incubation with γH2AX primary antibody at 1:1000 occurred overnight before embryos were washed in PBST and the secondary antibody (Alexa 488, goat anti-rabbit) was added in blocking buffer at 1:2000 for 2 h at 4°C in the dark (Table 2). Embryos were washed with PBST before being mounted in Vectashield Mounting Medium (VectorLabs, H1200-10) and imaged using an Olympus confocal microscope. Image analysis was performed using ImageJ software.

### Adult zebrafish locomotion

Five wild-type and five *rnaseh2a^−/−^* adult zebrafish were placed in single tanks and placed in front of the Adult Viewpoint Video system (*Viewpoint Behaviour Technology*) for 4 h. Tanks were taped between fish to prevent any shoaling influence on their movement. The first 1 h was treated as habituation time and discarded. This was repeated twice resulting in *n* = 10 for each genotype. Total distance moved via video tracking was calculated using Microsoft Excel 2016.

### Embryonic photomotor response

5dpf embryos were placed in individual wells of a 96-well plate (Corning® 3595) with 400 μl E3 medium. The embryos were transferred into the ZebraBox Viewpoint system (*Viewpoint Behaviour Technology*) and left to acclimatize for 1 h. The embryos were then subjected to three cycles of 5 min of light (80%) followed by 5 min of darkness (0%) and the total distance travelled by each embryo was quantified. Analysis of total distance moved in each light/dark cycle was performed using Microsoft Excel 2016.

### WGS analysis for detection of deletions

DNA was isolated using the Qiagen Blood and Tissue extraction kit following the manufacturer's instructions from 30 MZRnaseH2a embryos and 30 wild-type embryos, these were produced from a homozygous mutant or wild-type parents, all parents were brothers and sisters. Sequencing was performed by Novogene, and reads are available under accession number PRJEB65986 at ENA (https://www.ebi.ac.uk/ena/browser/home). Quality control checks of the FASTQ data was investigated using FastQC (Andrews, 2010). The reads were then aligned to the *Danio rerio* GRCz11 reference genome using bwa-mem2 and the reads that had a mapping quality score <20 were excluded from downstream analysis (Md *et al.*, 2019). Several tools from GATK were used for the subsequent data processing steps (36). The FASTQ files were then converted to unaligned BAM format using FastqToSam. The BAM files were then query-sorted using the SortSam tool and then re-written with new adapter-trimming tags using MarkIlluminaAdapters (Picard) tool. The MergeBamAlignment tool was then used to merge the read metadata. The PCR and optical duplicates were then marked using the MarkDuplicates tool. The dbSNP variants that were used as known sites for the base quality score recalibration were obtained from NCBI. GATK’s BaseRecalibrator followed by ApplyBQSR were used for the base quality score recalibration. Variant calling was then conducted using Mutect2 (37), SvABA (38) and bcftools call (39). For Mutect2, somatic calling of variants was then conducted using FilterMutectCalls. For all the variant calling tools, the default settings were used and only single files were used as inputs. To extract the indels after variant calling with bcftools call, the bcftools filter tool was used with TYPE=’INDEL’. The bcftools norm tool was then used to normalize the variants called and left align any ambiguous alignments. To identify the high confidence variants that were present in the output of at least two out of the three variant callers, the bcftools isec was utilized. The CHROM, POS, REF and ALT columns from the VCF files were extracted using bcftools query and written to a CSV file. The file was then imported in R (v 4.1.3) (https://www.r-project.org) where the difference in length between the alternate allele and reference allele was calculated and the frequency of indel events was determined and plotted using GraphPad Prism 9. Data manipulation in R required the readr (https://readr.tidyverse.org), and tidyr (https://tidyr.tidyverse.org) libraries.

### Statistical analysis

Graphs and statistical analysis were generated and performed using GraphPad Prism (Graphpad Software). Unpaired *t*-test or two-way ANOVAs were used as indicated in the figure legend with data presented as mean ± SD. *P*-values are indicated as follows: not significant (ns), *P*> 0.05; * *P <*0.05; **P <0.01; ***P <0.001 and ****P <0.0001. Three biological repeats were performed unless otherwise stated.

### Material availability statement

All data needed to evaluate the conclusions in the paper are present in the paper and/or the Supplementary Materials. The data underlying this article will be shared on reasonable request to the corresponding author.

## Results

### *rnaseh2a^−/−^* zebrafish are viable to adulthood

**Figure 1.**
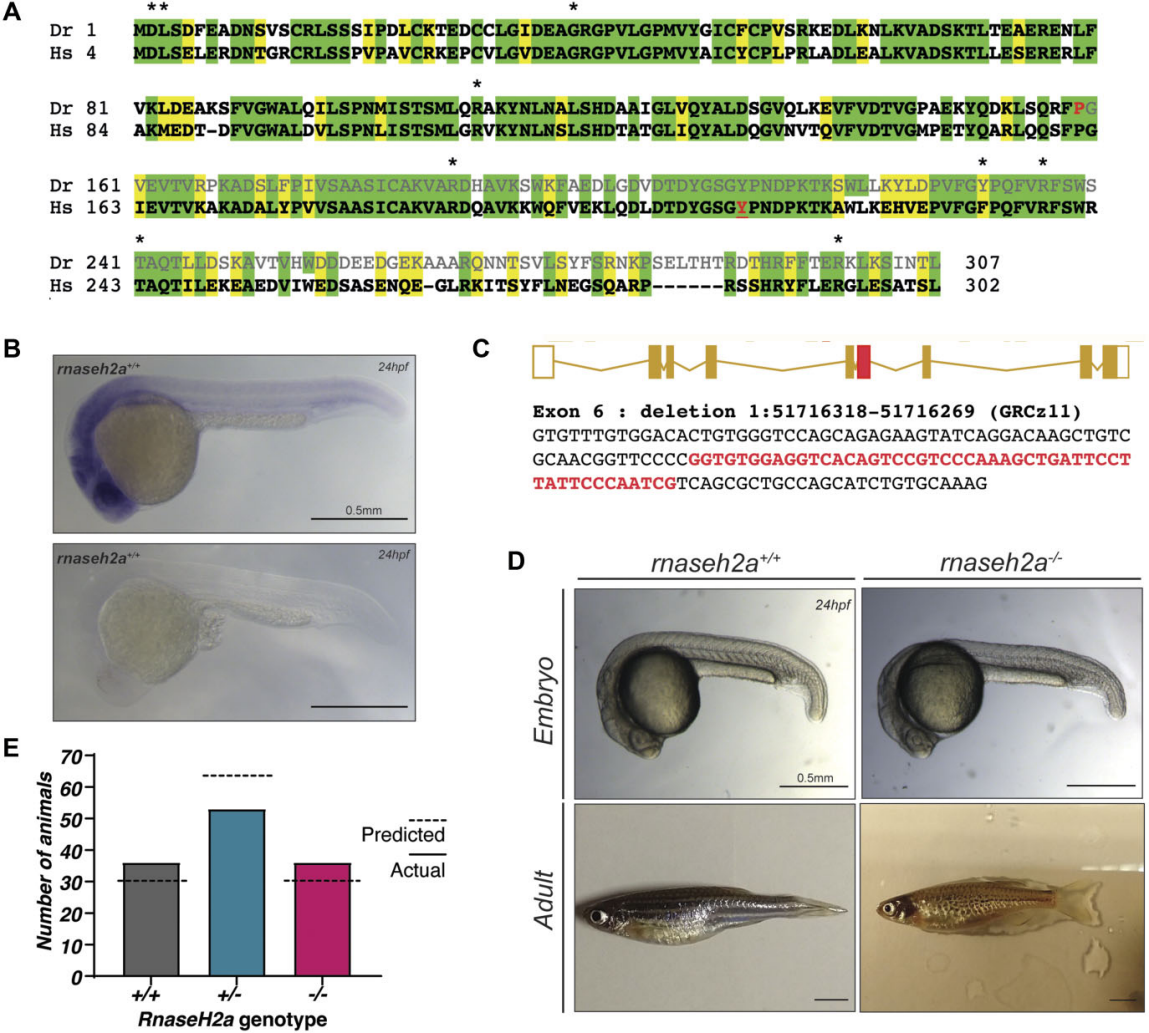
Generation of *rnaseh2a^−/−^* knockout zebrafish and initial characterization. (**A**) Protein alignment between zebrafish, *Danio rerio* (Dr) and *Homo sapiens* (Hs) RNaseH2a. In the zebrafish protein, the amino acids that are lost as a result of the mutation are greyed out, the last homologous amino acid is highlighted red. Asterisk show sites of human AGS mutations, a critical tyrosine required highlighted in red. (**B**) WISH staining shows universal RNaseH2a expression in wild-type zebrafish embryos at 24hpf, lower image shows sense probe as control. (**C**) In exon 6 (red), a 49bp deletion was created utilizing the CRISPR/Cas9 system. Exon 6 sequence is shown below with deleted bases in red. The deletion results in a premature stop codon and truncated protein of 205aa in length. (**D**) Embryos resulting from an *rnaseh2a^+/−^* incross do not show any visible phenotype and are able to develop to adulthood with no external phenotype. *NB*. spotty pigment is due to a background mutation in laboratory zebrafish. (**E**) Inheritance of the *rnaseh2a* mutation is in a homozygous recessive manner at 24hpf and displays mendelian inheritance. X^2^ = 2.66, with two degrees of freedom; two-tailed *P* value of 0.529.

As with humans, zebrafish contain a single ortholog of the Ribonuclease H2, subunit A (*rnaseh2a)*. The two have a high level of similarity at both the amino acid, and structural levels with zebrafish producing a protein of 307 amino acids (aa) (Figure 1A). *Rnaseh2a* was shown to have universal expression throughout wild-type zebrafish embryos at 24 h post fertilization (hpf) with a particular emphasis in the head (Figure 1B) which is in keeping with the expression profile shown in mice (40).

Using the CRISPR-Cas9 system, a 49bp deletion was created in Exon 6 (Figure 1C), this predicts a protein where approximately the first half (1–159aa) of Rnaseh2a is encoded, followed by a c-terminal tail of 46 unrelated amino acids (Figure 1A). This is a predicted null mutation, as it removes a highly conserved sequence, including amino acids that have been shown to be essential for RNaseH2a function in human (41) and also includes a critical tyrosine (Y211 in zebrafish) that is predicted to contact the free hydroxyl group on the ribonucleotide (42). Founders containing this deletion were outcrossed to wild-type zebrafish to create a *rnaseh2a^+/-^* line. Two *rnaseh2a^+/−^* fish were incrossed to create a stable *rnaseh2a^−/−^* line. Surprisingly, *rnaseh2a^−/−^* zebrafish appeared to have no obvious external phenotypes compared with their *rnaseh2a^+/+^* siblings at both embryonic and adult stages (Figure 1D) and were born at Mendelian ratios (Figure 1E). This is in contrast to all other null animal models targeting RNaseH2 created thus far in which all show early embryonic lethality (18,19,22).

### Offspring of *rnaseh2a^−/−^* have severe developmental defects

**Figure 2.**
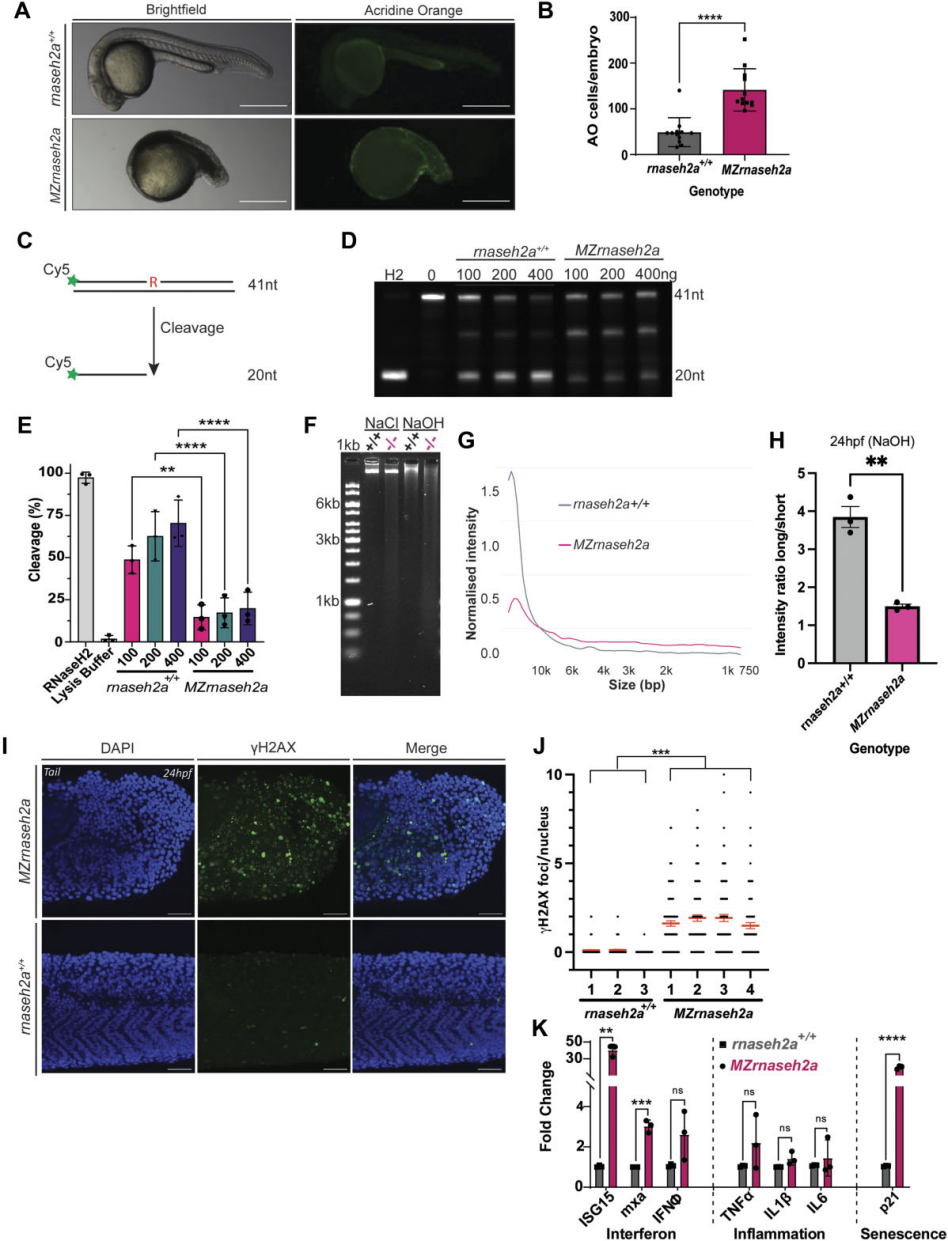
Offspring of *rnaseh2a^−/−^* adults have severe developmental defects, increased DNA damage, increased ribonucleotides and an upregulated inflammatory response. (**A**) Acridine Orange staining of apoptotic cells (green) in 24hpf embryos from homozygous and wild-type incrosses. (**B**) Quantification of acridine orange positive cells from (A). Unpaired *t*-test, ****P <0.0001, ±SD (*n* = 12, single embryo). (**C**) Schematic of single ribonucleotide cleavage assay. (**D**) Whole embryo lysate from 24hpf embryos were incubated with dsDNA substrate containing a single ribonucleotide. Positive and negative controls were purified RNaseH2 and lysis buffer respectively. DNA was tagged with Cy5. (**E**) Quantification of cleavage (%) in activity assay, ±SD, one-way ANOVA, *P <*0.05 (*n* = 3, 20 pooled embryos). (**F**) Gel of alkaline (NaOH) and control (NaCl) treated DNA of 24hpf embryos from homozygous and wild-type crosses. (**G**) Densitometry plot of DNA intensity produced with R. (**H**) Image quantification showing ratio of long and short fragments. Student's *t*-test **P< 0.01, ± SEM, *n* = 3. (**I**) γH2AX staining (green) in tails of 24hpf *rnaseh2a*^−/−^ embryos. Nuclei stained with DAPI (blue). (**J**) Quantification of γH2AX foci per nucleus in tail tip of individual embryos (>100 nuclei per embryo, 3 or 4 per genotype shown). Red lines show average per embryo and SEM. Unpaired *t*-test, Welch's correction, ***P <0.001, *n* ≥ 3). (**K**) qPCR of interferon stimulated genes (ISG15, mxa, IFNΦ), proinflammatory response markers (TNFa, ILb, IL6) and senescence markers (p21). ****P <0.0001, ***P <0.001, **P <0.01, ±SD (*n* = 3, 20 pooled embryos).

Despite the ability of *rnaseh2a^−/−^* from heterozygous parents to survive, the resulting offspring from a *rnaseh2a^−/−^* incross (maternal-zygotic (MZ) *rnaseh2a* mutants, hereafter referred to as *MZrnaseh2a*) were embryonic lethal. The embryos were fertilised and underwent several hours of normal development before their growth slowed to reveal reduced head and tail growth at 24hpf and the development of a large quantity of apoptotic cells (Figure 2A, B). Firstly, we wanted to confirm that the *MZrnaseh2a* embryos were unable to remove rNMPs from their DNA. Due to the absence of antibodies that can detect zebrafish RNaseH2, we assessed the catalytic activity of RNaseH2, which is a more reliable readout of a loss of function of the mutated protein. We used a cleavage assay whereby we incubated lysate from the whole embryos with a dsDNA containing a single rNMP that is labelled with a fluorophore, hence allowing the monitoring of the products of RNaseH2 cleavage activity (35) (Figure 2C). It was found that the *MZrnaseh2a* embryos were unable to cleave rNMPs as efficiently as their *rnaseh2a+/+* counterparts (Figure 2D, E). If this is the case then, due to the lack of cleavage activity, it was predicted that the *MZrnaseh2a* embryos should also have increased rNMPs in their DNA. To answer this question an alkaline assay was implemented, incubating DNA from embryos in alkaline conditions to encourage spontaneous hydrolysis of the rNMPs due to their 5′OH group. It was observed that *MZrnaseh2a* embryos had a significantly larger quantity of smaller DNA fragments post treatment suggesting an increase in rNMPs compared with the wt embryos (Figure 2F–H). With large numbers of rNMPs in the genome comes the susceptibility to spontaneous hydrolysis and formation of both single and double stranded DNA breaks. To determine whether the increase in rNMPs in *MZrnaseh2a* translates into an increase of strand breakage the double strand DNA (dsDNA) break marker γH2AX was used. The tails of *MZrnaseh2a* embryos showed significantly more γH2AX foci than the wt tails, indicating an increased level of breaks in the DNA. The morphology of the *MZrnaseh2a* tails was also shown to be drastically underdeveloped (Figure 2I, J). Given the large number of dsDNA breaks and subsequent fragmentation due to the increase in rNMP incorporation, it was predicated that this may result in the movement of nuclear DNA into the cytoplasm, activating the cGAS-STING pathway and subsequently causing upregulation of interferon stimulated genes as seen in AGS patients. To test this, quantitative PCR (qPCR) was performed on a selection of genes measuring the interferon (ISG15, mxa, IFNO), inflammatory (TNFa, IL-6, IL1b) and senescence (p21) responses. It was found that the *MZrnaseh2a* embryos had significantly higher expression levels of inflammatory markers ISG15, mxa, and senescence marker p21, as compared to wild-type embryos (Figure 2K). This is consistent with cGAS/STING activation, although alternative ways of activation cannot be excluded.

### *rnaseh2a^−/−^* adults are viable but show an increased incorporation of ribonucleotides

**Figure 3.**
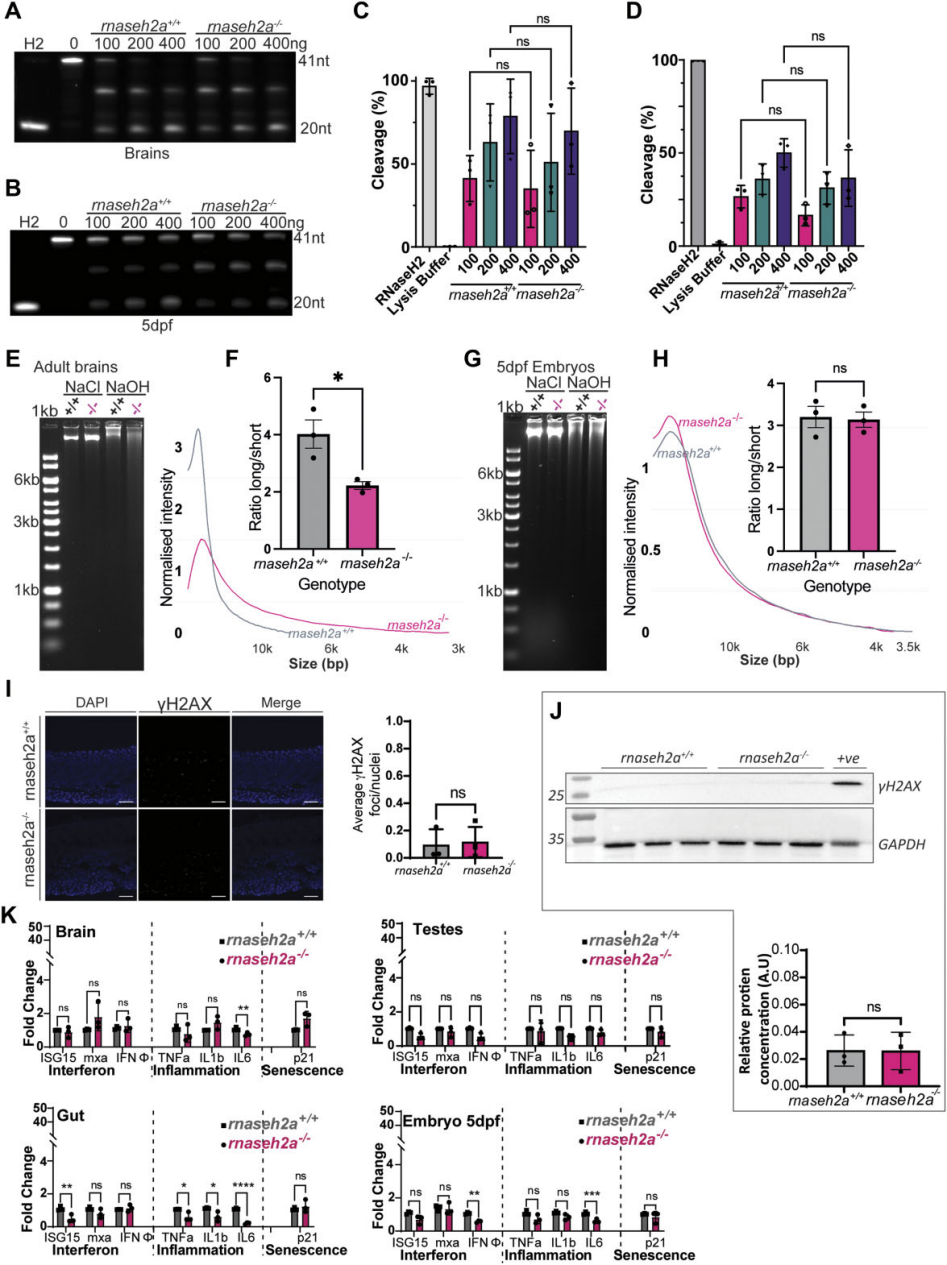
*rnaseh2a^−/−^* adult fish have a high incorporation of ribonucleotides but no increased inflammatory response. (**A**) RNaseH2 activity assay on brains from adult fish. (**B**) Activity assay of 5dpf embryos. (**C**) Quantification of cleavage assay on brains, ±SD, one way ANOVA, *P <*0.05, *n* = 3. (**D**) Quantification of cleavage activity of d5 embryos, ±SD, one way ANOVA, *P <*0.05, *n* = 3. (**E**) Alkaline treatment of DNA from brains from adults shows an increase of ribonucleotides in homozygote adults, as shown by smearing on a gel. (**F**) Quantification of alkaline treatment of adult brains. Image analysis shows that the ratio of long to short DNA fragments is significantly reduced in adult *rnaseh2a^−/−^* brains. Student's *t*-test, *n* = 3 ± SEM, *P< 0.05. (**G**) Alkaline treatment of DNA from on 5dpf embryos showing no increase in DNA smearing in homozygous embryos. (**H**) Quantification of alkaline treatment of 5dpf embryos. Student's *t*-test, *n* = 3 ± SEM. (**I**) γH2AX staining 5dpf embryo tails from a heterozygous incross. Quantification of foci per nuclei shows no significant difference between *rnaseh2a*^−/−^ and *rnaseh2a*^+/+^ siblings. Student's *t*-test, *n* = 3, *P <*0.05. (**J**) Western blot for γH2AX in brain samples from individual adult zebrafish. Quantification showed no significant difference between *rnaseh2a^−/−^* and *rnaseh2a^+/+^* siblings. Student's *t*-test, ±SD, *P <*0.05. *n* = 3. (**K**) Expression of interferon stimulated genes (ISG15, mxa and IFNphi), inflammatory response genes (TNFα, IL1β and IL6) and senescence markers (p21) were measured in tissues isolated from *rnaseh2a*-/- 19 month adults and in 5dpf *rnaseh2a*-/- embryos from a heterozygous incross. Multiple unpaired *t*-tests with Welch correction, *P <0.05, **P <0.01. ***P <0.001, ****P <0.0001, ±SD (*n* = 3).

As previously stated, *rnaseh2a^−/−^* from a heterozygous cross can survive to adulthood with no visible phenotypes (Figure 1D). To begin answering how they can survive, we first looked at their ability to cleave single rNMPs using the previously described cleavage assay. Unlike their offspring, *rnaseh2a^−/−^* adults were able to cleave the dsDNA substrate at equal efficiency to their wild-type siblings (Figure 3A, C). This suggests two possibilities, either there may be a very specific early embryonic requirement for RnaseH2a that is normally provided by maternal RNA or protein in the oocyte, or there is a secondary mechanism that has been upregulated (given the lack of RNaseh2a), and has taken over the removal of rNMPs. If there is such a secondary mechanism, we predicted that the *rnaseh2a^−/−^* adults would not have more rNMPs incorporated, and their DNA would behave like DNA from 5dpf zygotic *rnaseh2a-/-* larvae, not showing increased sensitivity to alkaline treatment. However, when subjected to alkaline conditions, the DNA extracted from brains of *rnaseh2a^−/−^* adults fragmented to a larger extent than brains from *rnaseh2a^+/+^* zebrafish (Figure 3E, F). This suggests that there is a slightly higher level of rNMPs incorporated into the *rnaseh2a^−/−^*, despite their cleavage ability. Given that adult tissues are much less proliferative than embryonic stages, we also wanted to check if this was the case at their embryonic stage, thus allowing a closer comparison with the phenotypes seen in their offspring. Using the cleavage assay once again, we were able to establish that at 5 days post fertilization (dpf), *rnaseh2a^−/−^* embryos from a heterozygous incross were able to cleave single rNMPs at an equal rate to their wild-type siblings (Figure 3B, D). This time, unlike during adulthood, we found that the level of DNA fragmentation was not increased in an alkaline assay, suggesting that there is no significant difference in the rNMPs incorporated into *rnaseh2a^−/−^* or wild-type zebrafish at 5dpf (Figure 3G, H). It appears therefore that although *rnaseh2a^−/−^* animals from a heterozygous incross maintain their ability to cleave single rNMPs, the incorporation of rNMPs increases from 5dpf to adulthood. Taken together, this suggests that although there may be a secondary mechanism involved in the removal of rNMPs, this process is not active in early development of *MZrnaseh2a* embryos, or is overwhelmed by a large number of inherited rNMPs in a manner that saturates the capacity of downstream repair factors such as FEN1/EXO1 and DNA ligase. Given the lack of rNMP incorporation into *rnaseh2a^−/−^* embryos from heterozygous parents, it was predicted that they would not show significantly more damage than their wild-type siblings. Once again, we used γH2AX staining and found that *rnaseh2a^−/−^* zebrafish at 5dpf contained no more dsDNA breaks than *rnaseh2a^+/+^*, confirming our hypothesis (Figure 3I). However, given the increase in rNMPs in the brains of adult *rnaseh2a^−/−^* zebrafish, we this time predicted that there would be a significant increase in *γ*H2AX in comparison with their wild-type siblings. After western blotting, it was clear that this wasn’t the case, with no significant increase between *rnaseh2a^−/−^* and *rnaseh2a^+/+^* brain samples (Figure 3J).

Given the lack of DNA damage and the absence of any significant phenotype, it was predicted that, unlike the *MZrnaseh2a*, the *rnaseh2a^−/−^* fish would not have an increased inflammatory response at either their adult or embryonic stages. Using qPCR it was found that in the adult brain, gut and testes there was no significant upregulation in any of the target genes. This was also the case at their embryonic stage (Figure 3K). Overall, this suggests that, despite having a mechanism that is able to remove single rNMPs from the genome, there is a slow accumulation over time but surprisingly this does not lead to a detectable increase in DNA damage, preventing early lethality.

### Resupplying RNaseH2a does not rescue embryonic lethality

**Figure 4.**
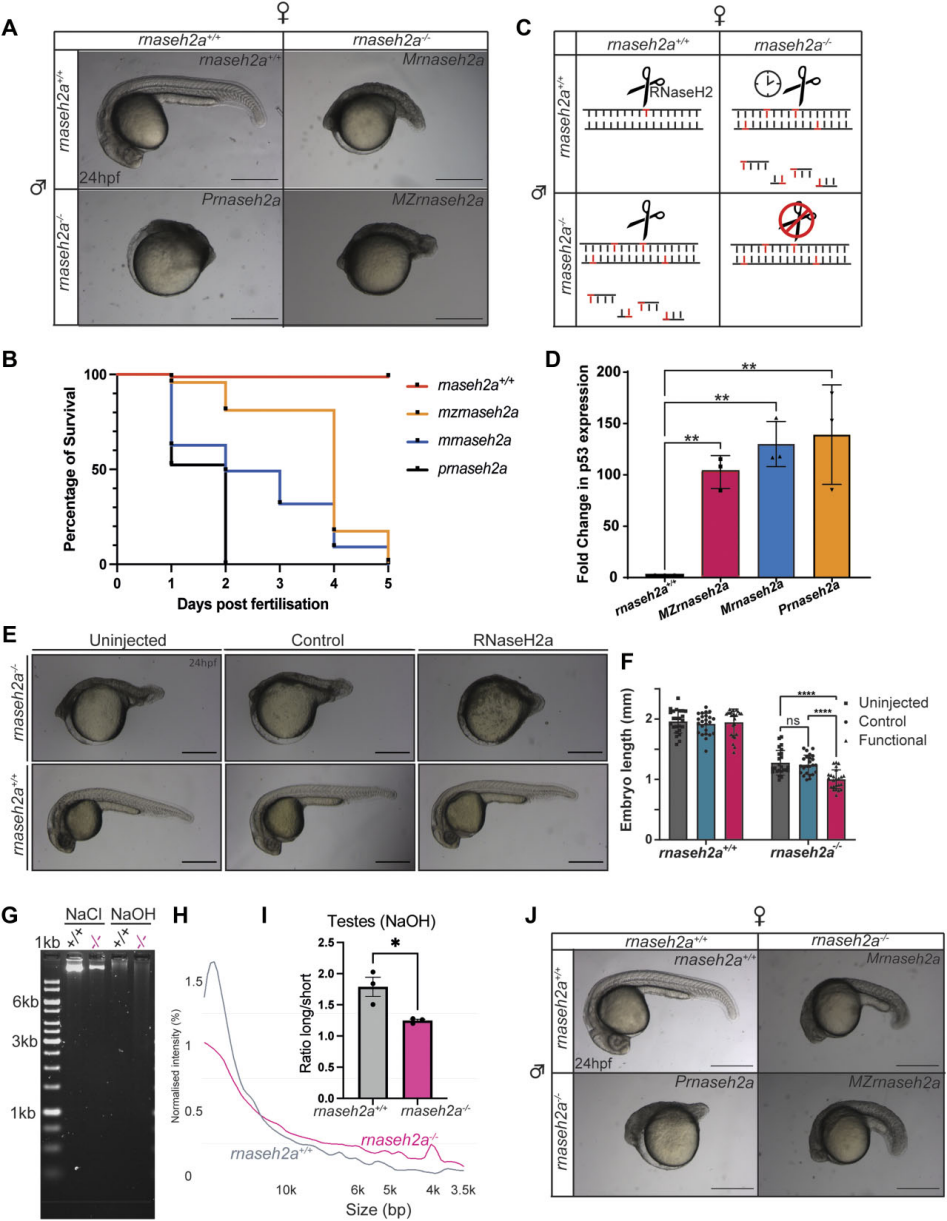
Re-introduction of RNaseH2a is unable to rescue the developmental phenotype. (**A**) Embryos resulting from various crosses with homozygous *rnaseh2a^−/−^* parents. (**B**) Survival curve of embryos with various homozygous parents (*n* = 110). (**C**) Schematic of predicted mechanism of detrimental phenotypes resulting from each parental cross. (**D**) RTqPCR analysis of a DNA damage sensitive p53 reporter (Δ113p53) shows significant upregulation of expression in all embryos resulting from a homozygote parent. ±SD, one-way ANOVA, **P <0.001 (*n* = 3, 20 pooled embryos). (**E**) Microinjection of RNaseH2a mRNA caused a more severe phenotype in embryos from a *rnaseh2a^−/−^* incross compared with the control or uninjected embryos. Embryos from a wild-type incross were uninjected. (**F**) Quantification of embryo size, ±SD, two-way ANOVA, ****P <0.0001 (*n* = 16). (**G**) Gel analysis of alkaline treated DNA from testes of adult males show an increase in ribonucleotides in *rnaseh2a*^-/-^ adult males. (**H**) Density plot of alkaline treatment of testes from adult zebrafish. (**I**) Ratio of long to short DNA fragments is significantly reduced in *rnaseh2a^−/−^* testes DNA after alkaline treatment. Student's *t*-test, *n* = 3 ± SEM, *P< 0.05. (**J**) Microinjection of a p53 morpholino visibly improves the development of all embryos with one or two homozygous parents (injected embryos are from same batch as the embryos shown panel A).

The zebrafish egg contains a lot of maternal products that support early development. Given the severe developmental phenotypes displayed by 100% of the offspring produced by two *rnaseh2a^−/−^* adults, we expected this to be a maternally contributed phenotype. To check this, we crossed a *rnaseh2a^−/−^* male with a *rnaseh2a^+/+^* female, expecting normal embryos. Surprisingly, it was found that these heterozygous embryos (Paternal *rnaseh2a*, hereafter referred to as *Prnaseh2a*) were developmentally, significantly worse than the *MZrnaseh2a* embryos (Figure 4A). We also found that a *rnaseh2a^−/−^* female crossed with a *rnaseh2a^+/+^* male produced offspring (maternal *rnaseh2a*, hereafter referred to as *Mrnaseh2a*) that were slightly more developed but still lethal by 3dpf (Figure 4B). MZ-, M- and Prnaseh2a embryos all show strong upregulation of a p53 DNA damage specific isoform (Δ113p53) (Figure 4D) (43). The displayed embryonic defects in all pairings can be delayed via the injection of a p53 morpholino, that is known to interfere with p53 expression (33,34). (Figure 4J compare with A). Although this does not totally rescue the embryo, it does allow them to develop beyond their original phenotypes, demonstrating the key involvement of apoptotic signalling in the survival of the embryos, and a likely involvement of DNA damage response in the phenotype.

We hypothesized that the accumulation of rNMPs in the testes and sperm in an *rnaseh2a^−/−^* male is subsequently inherited by the embryo. The sperm cell contributes only two essential elements to a zygote, a centrosome and their DNA. The sperm centrosome is essential to allow normal cleavage of the zygote, and this was unaffected (not shown). Therefore, the most likely cause of the abnormal development of paternal mutant embryos might reside in their DNA.

If the embryo has originated from a wild-type mother, it will already have an inherited, active RNaseH2a. This will recognize and cleave the ribonucleotides inherited from the male, leading to a large number of DNA damage events, more than it can repair, resulting in an upregulation of key apoptotic factors such as p53 and leading to cellular death. The same logic can be applied to the slightly healthier *Mrnaseh2a* embryos, resulting from a homozygous mother and wild-type male. This time however, the ribonucleotides are inherited from the mother and the DNA provided by the male is not actively transcribed in the zygotic embryos until ∼3 h post fertilization at the mid blastula transition. This means that, until the 1000 cell stage, the embryo has only minimal amounts RNaseH2a present as sperm is unlikely to contribute significant amounts of this *rnaseh2a* mRNA or protein (44,45). However, once this zygotically produced *rnaseh2a* mRNA is translated, it recognizes and cleaves the large number of ribonucleotides in the DNA of the embryo. Therefore, although it survives slightly longer than the embryos from a homozygous male, the large quantity of subsequent apoptosis remains lethal, however, just delayed (Figure 4B).

If this hypothesis is correct, the injection of active *rnaseh2a* mRNA would cause recognition of the large number of rNMPs present in the DNA and cause mass cleavage events, which are predicted to be significantly detrimental to the embryo, rather than increasing their survival. When mRNA for active *rnaseh2a* was injected into a 0 dpf embryo, at 24hpf there was a significant decrease in development in embryos compared with those that had been injected with a control, inactive *rnaseh2a*, or non-injection control (Figure 4E, F). This phenotype is not seen when mRNA encoding an active *rnaseh2a* is injected into homozygotes from a heterozygous incross, suggesting that the lack of inherited ribonucleotides allows them to survive with no visible phenotype. To further confirm this, we used the alkaline assay to check for rNMP levels in the testes of *rnaseh2a^−/−^* adult males. It was found that, although there is a general sensitivity to alkaline condition in the testes, those from *rnaseh2a^−/−^* fish were more sensitive, resulting in a higher number of shorter DNA fragments, concurrent with an increased number of rNMPs (Figure 4G–I). This suggests that if the number of rNMPs incorporated into their gametes is over a certain threshold then embryos, with their rapid cellular division, are not able to overcome the number of rNMPs once fertilized. This also supports the hypothesis that it is the introduction of active RNaseH2 into DNA with high numbers of rNMPs that causes the detrimental phenotype displayed by *Prnaseh2a* embryos. All of these results also corroborate a phenomenon found in yeast termed ribodysgenesis, the process of an active RNaseH2 being introduced to an environment containing high levels of rNMPs causing mass cleavage events (Figure 4J) (46).

### rNMP build up affects movement and reproduction

**Figure 5.**
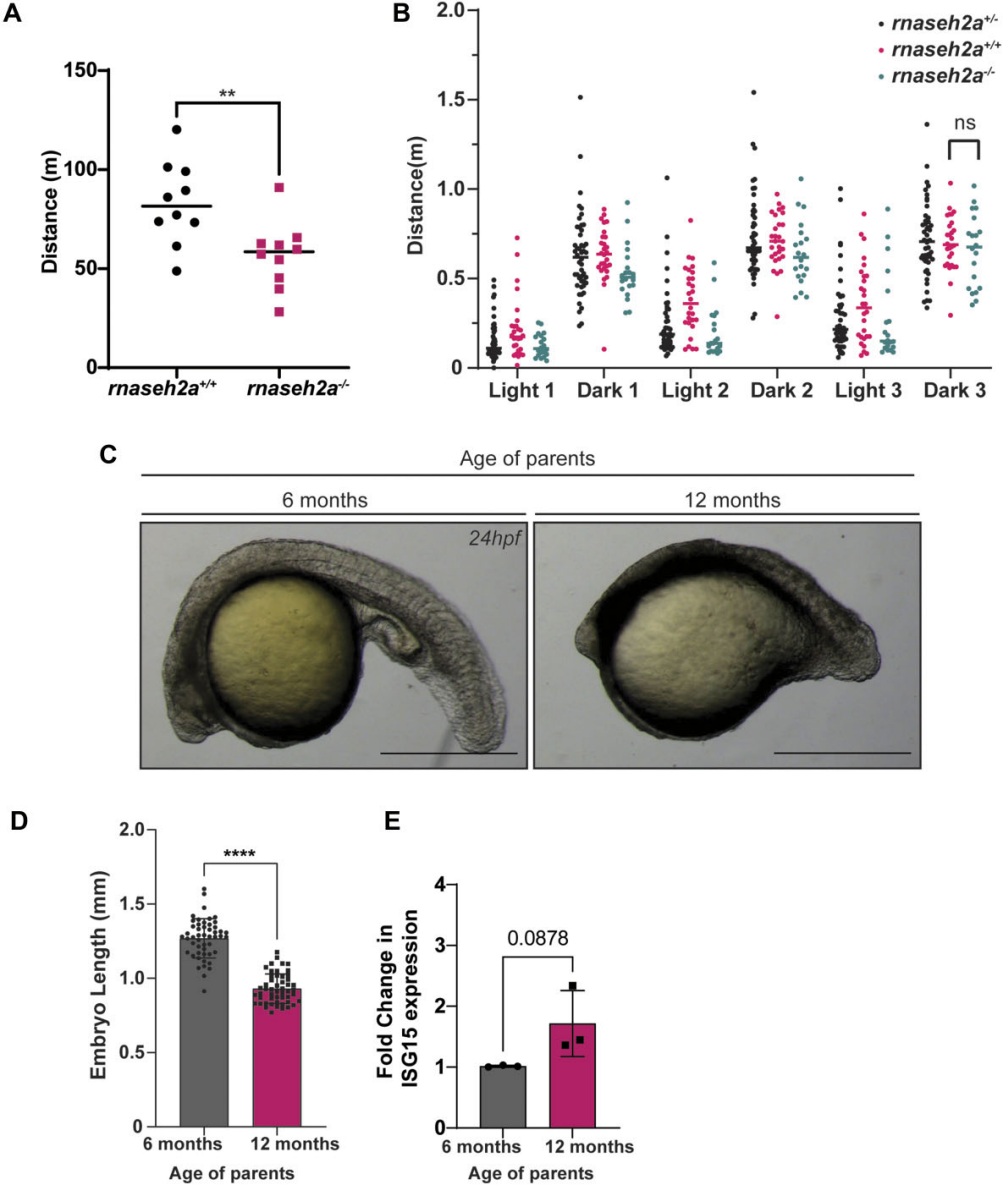
Embryos from older rnaseh2a^−/−^ fish are significantly underdeveloped compared with embryos from younger fish. (**A**) Movement analysis of adult zebrafish revealed the distance moved by *rnaseh2a*^−/−^ is significantly shorter than *rnaseh2a*^+/+^ adults. Unpaired *t*-test, *n* = 10, individual adults, ±SD, ** *P* < 0.001. (**B**) Photomoter response of embryos from *rnaseh2a*^+/−^ parents show no significant difference in distance moved by *rnaseh2a*^+/+^ compared with *rnaseh2a*^−/−^ ±SD, unpaired *t*-test, *n* = *P* < 0.05. (**C**) Representative images of embryos from 6 month old and 12 month old *rnaseh2a*^−/−^ zebrafish. (**D**) Quantification of embryo length from old and young zebrafish *n* = 36, individual embryos. Unpaired *t*-test **** *P* < 0.0001. (**E**) RTqPCR showing an increase in the expression level of ISG15 in embryos resulting from older *rnase2a*^−/−^ adults. *n* = 3, 20 pooled embryos. Unpaired *t*-test *P* < 0.05.

Whilst the adult *rnaseh2a^−/−^* zebrafish remain without any obvious morphological phenotypes, they were shown to have increased rNMP incorporation in their brains, and in the testes of males. To determine whether this leads to more subtle defects, we measured their movement over 3 h (after 1hour acclimatization to the measurement setup) using video tracking. It was found that total distance that 15 month old *rnaseh2a^−/−^* moved in that period, was significantly less than their *rnaseh2a^+/+^* siblings (59 versus 82 m on average) (Figure 5A). Again, to check if this phenotype was present at a younger age, 5dpf embryo were analysed. This time, no difference was seen in the distance moved by *rnaseh2a^+/+^* compared with *rnaseh2a^−/−^*(Figure 5B). This suggests that the increase in rNMP incorporation over time from 5dpf to adulthood may also influence their locomotion, a clinical characteristic of AGS patients, however the precise link between these remains unclear.

If this increase in rNMPs is occurring from 5dpf to adulthood, it could also continue throughout adulthood, and perhaps lead to more severe defects later in life. This was supported by the discovery that *MZrnaseh2a* embryos from parents that were 12 months old, were significantly less developed than those from parents that were only 6 months old (Figure 5C, D). Along with reduced development, *MZrnaseh2a* embryos from older parents also displayed an increase in ISG15, one of the key interferon-stimulated genes seen upregulated in *MZrnaseh2a* embryos (Figure 5E). Overall, these observations support the prediction that, if there is a secondary mechanism removing rNMPs when RNaseH2 is not active, it is inefficient. This may lead to increasing ribonucleotide levels in the DNA as the fish ages and increased inheritance of rNMPs by embryos from older parents, leading to further detrimental phenotypes.

### *rnaseh2a^−/−^* embryos are not sensitive to knockdown or inhibition of TOP1

Given the ability of *rnaseh2a^−/−^* zebrafish from heterozygous parents to survive, it is predicted that there may be secondary mechanisms that are compensating for the lack of RNaseH2 activity. The prime candidate for a secondary repair pathway is through Topoisomerase 1 (TOP1) activity (47). The primary role of TOP1 is the relief of torsional stress by cleavage of a single DNA strand. After cutting, TOP1 becomes covalently bound to the DNA, resulting in the formation of a TOP1 covalent complex (TOP1-cc). This complex is resolved by reversal of its covalent binding, and subsequent religation of the break (48).

In the absence of RNaseH2, TOP1 removal of rNMPs occurs in an error-prone manner through the cleavage of an rNMP creating a non-ligatable nick before cleaving for a second time further upstream. This can result in strand realignment and the creation of an ‘ID4-signature’ where insertion-deletion (indels) of 2–5 bases are prevalent (49). This is a key mutational hallmark of many cancers (49,50). To interfere with TOP1 function we co-injected guides targeting both Top1 and Top1L with Cas9 protein, we found that *rnaseh2a^−/−^* embryos from a *rnaseh2a^+/−^* cross were not significantly more affected than their *rnaseh2a^+/+^* siblings (Supplementary Figure S1A–C). Thus, TOP1 is unlikely to be crucial for survival of *rnaseh2a^−/−^*. Furthermore, if TOP1 is primarily responsible for the removal of rNMPs in the absence of RNaseH2, it would be expected that stalling of the TOP1-cc complexes via the TOP1 inhibitor, camptothecin (CPT) would result in *rnaseh2a^−/−^* zebrafish being more sensitive than their wild-type siblings. When treated overnight with CPT, the resulting developmental phenotypes seen in an *rnaseh2a^+/−^* incross are not increased in *rnaseh2a^−/−^* embryos after blind selection and genotyping (Supplementary Figure S2A). Altogether these experiments indicate that TOP1 is not essential to compensate for loss of RnasH2a function in zebrafish larvae.

If TOP1-mediated removal is occurring in *MZrnaseh2a* embryos which are devoid of RnaseH2 function, high-level TOP1-mediated DNA cleavage events may in fact be perceived as DNA damage, and *cause* poor development and cell death. If this is the case, creation of a TOP1 CRISPANT in *MZrnaseh2a* embryos might alleviate their lack of development. However, we found that *MZrnaseh2a; top1* CRISPANT embryos showed no significant change in development compared with their uninjected siblings (Supplementary Figure S1D, E, G), nor did we see an increase in TOP1 expression in *MZrnaseh2a* embryos (Supplementary Figure S1F). Whole genome sequencing of *MZrnaseh2a* embryos also showed no significant increase in 2–5 bp indels, which is typical of the ‘ID4-signature’, compared with *rnaseh2a^+/+^* embryos (Supplementary Figure S1H).

Genome wide screens have also shown synthetic lethality of *rnaseh2a^−/−^* cell lines with the inhibition of PARP or ATR (51,52). In zebrafish however, we show that treatment with an ATR inhibitor (Supplementary Figure S2B) that showed phenotypes consistent with increased DNA damage (small head and eyes), as expected, had no significantly detrimental impact on *rnaseh2a^−/−^* embryos compared with their wild-type siblings. Similar observations were noted for PARP inhibitors (Supplementary Figure S2C). This suggests that there are further mechanisms responsible for the removal of rNMPs in the absence of RNaseH2. We did not observe sensitization of *rnash2a^−/−^* embryos to hydroxyurea, which increases the rNMP/dNMP ratio (Supplementary Figure S2D). Finally, we tested a potential role of RnaseH1 in preventing zygotic phenotypes from occurring, however knockdown of this gene using 2 guides did not produce a detectable phenotype in *rnaseh2a* mutants or their siblings (Supplementary Figure S2E).

## Discussion

Several animal models have been produced to study the role of the three RNaseH2 subunits in disease. A common phenotype that they all shared was early embryonic lethality (18–22). This was determined to be due to the build-up of rNMPs, increased DNA damage and subsequent induction of the Type 1 interferon response (2,4,5).

Here, we have produced an *in vivo* knockout zebrafish model of *rnaseh2a*. Unlike previous models, this *rnaseh2a^−/−^* zebrafish is viable to adulthood showing no overt physical phenotypes apart from a mild reduction in locomotion which is not present at an embryonic stage. However, whilst reaching adulthood, *rnaseh2a^−/−^* zebrafish appear to accumulate an increased number of rNMPs into their genome, not present at 5dpf, at least in their testes and their brains as judged by the ratio of long/short genomic DNA fragments, after alkaline digestion.

It is in principle possible that a maternal contribution of RNAseH2 protein or mRNA in F1 zygotic mutants is responsible for allowing their continued development, but as maternal mRNAs are also actively removed after mid blastula transition (MBT) (53,54), it is difficult to understand how a very large amount of cell division post-MBT can take without alternative ways to remove mis-incorporated RNAs. We therefore prefer the idea that, upon the removal of RNaseH2a, there is a secondary mechanism that removes rNMPs albeit much less efficient than RNaseH2, and therefore is slowly overwhelmed by their continued incorporation. This secondary mechanism may contribute to the cleavage activity in our rNMP oligo-assay with RNaseH2a deficient extracts from 5dpf larvae and adults. It also suggests that the mild build-up of rNMPs may be responsible for the reduced motor response in adults, similar to the detrimental locomotion phenotypes seen in AGS patients.

Secondary repair mechanisms in the absence of RNaseH2 have previously been described. The main candidate being Topoisomerase 1 ‘TOP1’ (55). TOP1 is able to remove rNMPs in the absence of RNaseH2 but has recently been shown to do so in an error prone manner (49,50). However, interfering with the catalytic cycle of TOP1 by the use of the TOP1 poison, camptothecin ‘CPT’ did not significantly affect *rnaseh2a^−/−^* embryos, nor did it rescue *MZrnaseh2a* developmental phenotypes. Coupled with their insensitivity to ATRi and PARPi, previously shown to cause synthetic lethality with knockout of RNaseH2 due to accumulation of TOP1-driven toxic repair intermediates (51,52,56), it does not appear that TOP1 activity is the main driver behind the survival of RNaseH2 deficient animals. Although we did not find a measurable evidence that TOP1 is essential, we cannot rule out the possibility that TOP1 may still be present and active, and may be partially responsible for the cleavage activity that we observed in our single ribonucleotide cleavage assay.

The main surprise however was the embryonic lethality displayed in any offspring resulting from all *rnaseh2a^−/−^* adults. The severity of the phenotype increased from *MZrnaseh2a* to *Mrnaseh2a* with *Prnaseh2a* embryos dying earliest at 24hpf. All offspring show a large upregulation in p53 expression and *MZrnaseh2a* embryos indicate a large upregulation in interferon stimulated genes such as ISG15. The early lethality of *MZrnaseh2a* embryos is suggested to be due to the large build-up of rNMPs in their genomic DNA, resulting in instability and spontaneous cleavage. The severe phenotypes of *Mrnaseh2a* and *Prnaseh2a* embryos are predicted to be the result of ribodysgenesis, where an active RNaseH2a comes into contact with a large number of incorporated rNMPs (46), resulting in a large number of repair intermediates that cannot be fully processed because they overwhelm downstream repair steps. However, further experiments would be necessary to prove this idea, for instance by downregulating maternal RNAseH2 in *Prnaseh2a* embryos or overexpression of FEN1/EXO1 and DNA ligase to see if the phenotype is rescued. Our data is supported by the increase of rNMPs seen in the testes of adult males suggesting the inheritance of a large number of ribonucleotides by their offspring. We have also found that this phenotype becomes more severe in the offspring from older adults, suggesting a steady increase of rNMPs in the gametes of *rnaseh2a^−/−^* adult zebrafish.

In summary, we have created an *rnaseh2a^−/−^* zebrafish line which is viable to adulthood despite showing an accumulation of rNMPs from embryonic stages through to adult. These rNMPs are inherited by any offspring from two *rnaseh2a^−/−^* parents causing genome instability and early embryonic lethality. We show that this phenotype increases in severity with the addition of a *rnaseh2a^+/+^* parent due to the addition of an active RNaseH2 into a high rNMP background. This finding is exciting due to no previous *in vivo* RNaseH2a knockout being viable past embryonic stages and the stark difference between adults and offspring of the same genotype. We propose a mechanism of rNMP incorporation over time in *rnaseh2a^−/−^* zebrafish and the subsequent inheritance of the accumulated rNMPs causes early embryonic lethality. The severity of which increases with the age of the parents. We also predict a secondary mechanism of rNMP removal that allows the survival of *rnaseh2a^−/−^* that is not reliant on TOP1, this was further confirmed by whole genome sequencing that did not show the expected ID4 signature. Therefore, the RNaseH2a mutant provides a sensitized background that will aid the identification of the molecular nature of this alternative RNA removal mechanism.

## Supplementary data

Supplementary Data are available at NAR Online.

## Supplementary Material

gkae725_Supplemental_Files

## Notes

Present address: Arwa A. Abugable, Department of Biochemistry, University of Oxford, South Parks Road, Oxford, UK.
