# Supplementary material for: Second generation lethality in RNAseH2a knockout zebrafish
Source: Nucleic Acids Res. 2024 Sep 1;52(18):11014–28. doi: 10.1093/nar/gkae725 (PMC11472149; doi:10.1093/nar/gkae725)
Supplement: gkae725_Supplemental_Files [file gkae725_supplemental_files.zip › Supplementary Methods NARtest.docx]

**Supplementary Methods**

**Procedure for determining DNA High/Low molecular weight ratios on alkaline gels**

In imageJ, a standard box was drawn around the high and low molecular weight DNA and a ratio of average greyscales was determined. An example is shown in the sample image below. The same boxes were reused for all lanes. All biological repeats were run on a single agarose gel.


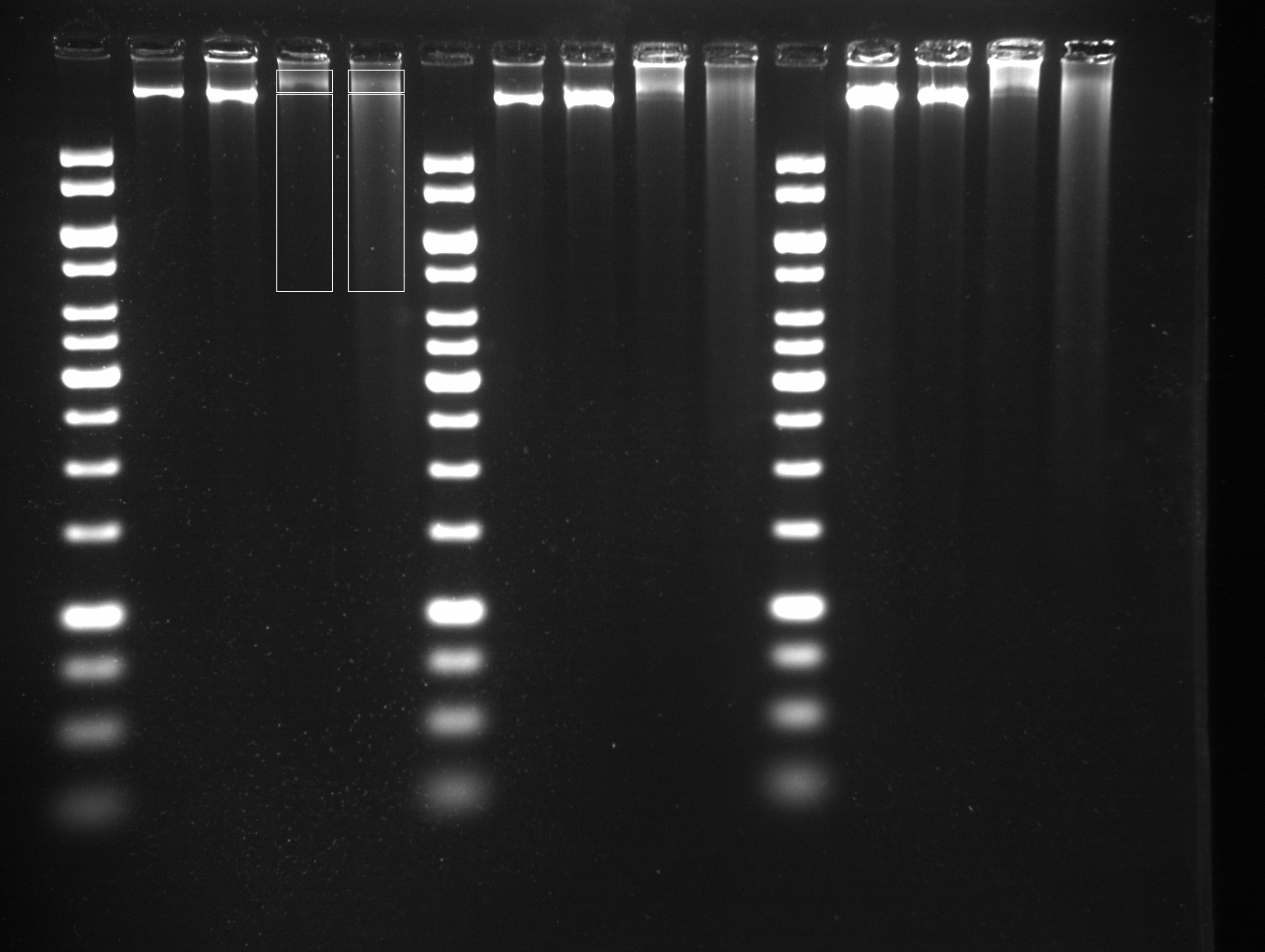


**Procedure for plotting DNA profiles after NaOH treatment (and controls)**

Image files showing a single gel with all three biological replicates of NaCl and NaOH-treated wildtype or *rnaseh2a* mutant DNA were opened in **Fiji.** The image was rotated until the slots were perfectly vertical, with the DNA and the ladder running horizontally to the right. Then a box was drawn that covered the lane with the ladder. This box was saved using *Edit>Selection>Save to Manager* and used for all measurements, (the box was only moved in the Y-direction, to cover lanes that needed measurement).

While the box was positioned over the DNA ladder, the *Tools>Plot Profile* function (using standard settings) was used, to obtain the greyscale values from the ladder along the x-axis. These were displayed using the “List” button in the Plot window, resulting in opening of a “Plot Values” window.

Next, an **Excel** file was created with 3 tabs: *Data, Blank, Ladder*.

In the “*Data*” tab, add the following column labels in the first row: Distance Set Treatment Type Value. (see **Fig 1** for an example)

In the “*Blank*” tab, two column labels are needed in the first row : Set Value (see **Fig 2** for an example)

In **FIJI,** in the “*Plot Values*” window, the “*Gray_Value*” column of ladder values was copied, (without any column label) in the “*ladder tab*” of the **Excel** file. Then it is identified which excel row value corresponds to each peak in the ladder peak; use *Insert>Chart>Line* in **Excel** to create a chart that can be used help with this. This will result in a list of DNA size values from the ladder, with a corresponding list of row values, for instance

"10k", "8k"," 6k", "5k", "4k", "3k", "2k", "1.5k", "1k", “0.5k”

and

98, 127, 172, 208, 252, 281, 313, 356 407 472

An **R script** was written to create an average profile of the three biological repeats for each genotype and treatment. The R script needs to “know” where the ladder values are for printing DNA sizes on the x-axis of a density plot. The serial row numbers that were determined above from the Excel file are used to do this. However, in order to do this correctly the excel row numbers determined above need to have 2 subtracted from them…(Using the sample above. They would need to be 96, 125, 170, 206, 250, 279, 311, 354, 405, 470)

These numbers need to be entered in the R script (see below) on line 60, the corresponding ladder size values need to be entered in line 61, the same needs to be done on line 85 and 86

Next in **FIJI**, the DNA intensity values are determined for the first experimental DNA lane using the saved box, it is moved only using up of down arrow keys. Again, the *Tools>Plot Profile function* is used, followed by the “*List*” button in the Plot window, resulting in opening of a “Plot Values” window, and the Gray_Value” column of values was copied and pasted into the “Value column” of the “Data tab” of the **Excel** file. Then the other columns of this tab are filled: The Distance will be filled with 0, 1,2,3,4…etc until the last available value. The “Set Column” refers to the biological repeat and will be filled with 1 for the first repeat, 2 and 3 for the second and third repeat. The “Treatment” column will be NaCl (control) or NaOH (experimental) The “Type” column will be the genotype: either wild-type or *rnaseh2a.* An example of the head of a file is shown below in **Fig 1**.


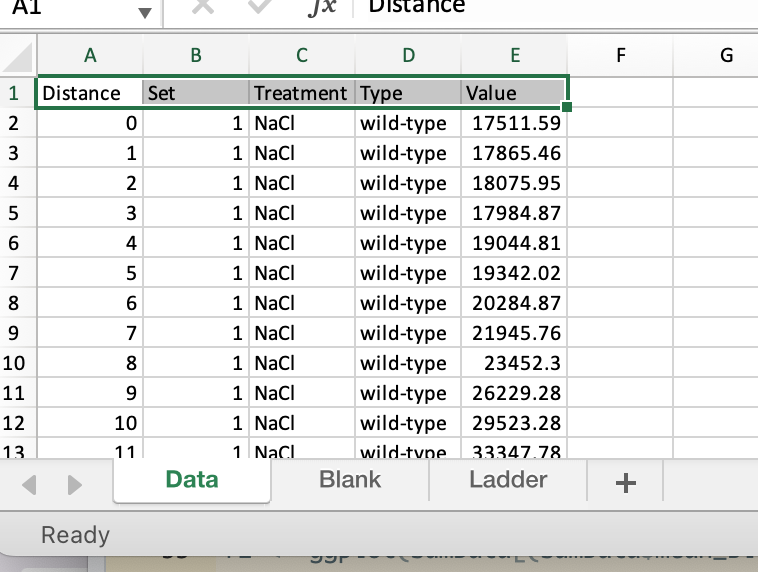


Then the next lane is measured in the same way, and the values are pasted ***below*** the existing values. Again, distance needs to start at 0,1,2,3,4….etc.

This procedure is continued until all lanes are measured, thus this will lead to a *very long* list 5 columns wide.

Finally, a background value needs to be determined, this can be done by drawing very thin boxes that cover the gel in between lanes, use three of these across the gel, the values from these will be used to create a single average background value. Again, plot profile is used, and “Gray_Value” column needs to be pasted in the Value column in the Blank tab of the **Excel** file, use values 1,2,3 for the different boxes.

After doing this save the **Excel** file you have created and close it, then find the precise path to that file, and copy that name to the clipboard


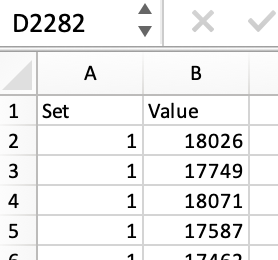


Below is the R script for plotting profiles

Before it can run the text on line 9 and 10 needs to be edited, **file path to excel file** needs to be replaced with the actual path that should be in the clipboard. (Previously, the ladder values on line 60+61 and 85+86 should have been edited) Now the script can be copied into eg R Studio and run.

library(tidyverse)

library(ggpubr)

library(cowplot)

library(readxl)

library(xlConnect)

## Data import ----

gel_val <- read_excel("**file path to excel file**", sheet = "Data")

blank <- read_excel("**file path to excel file** ", sheet = "Blank")

## Looking at data ----

str(gel_val)

str(blank)

## Plotting the data ----

ggplot(gel_val, aes(x = Distance, y = Value, colour = Type))+

geom_point()+

theme_bw()

## Calculating blank ----

mean_blank <- mean(blank$Value)

mean_blank

## Subtracting blank from data ----

gel_val$Blanked <- apply(gel_val[c("Value")], 1, function(x){x - mean_blank})

str(gel_val)

## Summarizing data ----

sumData <- gel_val %>%

group_by(Distance, Treatment, Type) %>%

summarise(

mean_Value = mean(Value, na.rm = TRUE),

sd_Value = sd(Value, na.rm = TRUE),

se_Value = sd(Value, na.rm = TRUE)/sqrt(sum(!is.na(Value))),

mean_Blanked = mean(Blanked, na.rm = TRUE),

sd_Blanked = sd(Blanked, na.rm = TRUE),

se_Blanked = sd(Blanked, na.rm = TRUE)/sqrt(sum(!is.na(Blanked)))

)

sumData

## PLOTTING DATA ----

## Plotting summery data with blanking ----

# Y axis removed for final figure

f1 <- ggplot(sumData[(sumData$mean_Blanked>0),], aes(x = Distance, y = mean_Blanked, colour = Type))+

geom_smooth(aes(group = Type), span = 0.1, size = 1.1, se = FALSE)+

facet_wrap(Treatment ~., scales = "free_y", nrow = 2)+

ylab("Intensity")+

xlab("Size (bp)")+

scale_x_continuous(breaks = c(96, 125, 170, 206, 250, 279, 311, 354, 405, 470),

labels = c("10k", "8k", "6k", "5k", "4k", "3k", "2k", "1.5k", "1k", "0.5k",))+ # Thermo scientific ladder

scale_colour_manual(values = c("NMR" = "red3",

"Mouse" = "deepskyblue3"))+

#"1109 days" = "red3",

#"860 days" = "darkorange1",

#"238 days" = "deepskyblue3",

#"180 days" = "slategray",

#"ladder" = "black"))+

theme_minimal()+

theme(legend.position = "bottom")+

theme(strip.text.x = element_text(size = 12, colour = "black", face = "bold"))+

theme(panel.grid.major.x = element_blank())+

theme(panel.grid.minor.x = element_blank())+

theme(panel.grid.major.y = element_blank())+

theme(panel.grid.minor.y = element_line())

f1

f2 <- ggplot(sumData[(sumData$mean_Blanked>0),], aes(x = Distance, y = mean_Blanked, colour = Treatment))+

geom_smooth(aes(group = Treatment), span = 0.1, size = 1.1, se = FALSE)+

facet_wrap(Type ~., scales = "free_y", nrow = 2)+

ylab("Intensity")+

xlab("Size (bp)")+

scale_x_continuous(breaks = c(96, 125, 170, 206, 250, 279, 311, 354, 405, 470),

labels = c("10k", "8k", "6k", "5k", "4k", "3k", "2k", "1.5k", "1k", "1k",))+ # NEB ladder

scale_colour_manual(values = c("NaOH" = "deepskyblue", "NaCl" = "darkslategray"))+

theme_minimal()+

theme(legend.position = "bottom")+

theme(axis.title.y = element_blank())+

theme(strip.text.x = element_text(size = 12, colour = "black", face = "bold"))+

theme(panel.grid.major.x = element_blank())+

theme(panel.grid.minor.x = element_blank())+

theme(panel.grid.major.y = element_blank())+

theme(panel.grid.minor.y = element_line())

f2

## Combining figures ----

# with blanking:

plot_grid(f1, f2, labels = c("A", "B", label_size = 12))
