# Supplementary material for: Second generation lethality in RNAseH2a knockout zebrafish
Source: Nucleic Acids Res. 2024 Sep 1;52(18):11014–28. doi: 10.1093/nar/gkae725 (PMC11472149; doi:10.1093/nar/gkae725)
Supplement: gkae725_Supplemental_Files [file gkae725_supplemental_files.zip › Supplementary Figures changeinblack.docx]

**Supplementary Figure 1: *rnaseh2a^-/-^* embryos are not more sensitive to G0 knockout of Top1 and Top1L**
A) Phenotypic images demonstrating no difference in severity between *rnaseh2a^+/+^,* *rnaseh2a^+/-^* and *rnaseh2a^+/-^* embryos upon microinjection of guides targeting TOP1 and TOPL. B) Quantification of genotypes in each phenotype. *rnaseh2a^+/+^* n=12, *rnaseh2a^+/-^* n=7, *rnaseh2a^-/-^* n=7, individual embryos C) Representative gel showing smearing of target-site PCR product after injection of TOP1 and TOP1L guides D) Microinjection of guides targeting TOP1 and TOPL (zebrafish duplication) show no significant difference in phenotype of *MZrnaseh2a* embryos at 24hpf. Representative images. E) Quantification of embryo length from B. Unpaired t-test ±SD, ns *P* >0.05, n= 12, individual embryos. F) Quantitative PCR of TOP1 and TOP1L expression in *MZrnaseh2a*  embryos at 24hpf. Unpaired t-test ±SD, ns *P*>0.05, n=3, 20 pooled embryos. G) Representative agarose gels showing smearing of wild-type band, indicating multiple cleavage instances of *top1 and top1L* H) WGS analysis of indels; no specific increase of 2-5bp deletions is observed in *MZrnaseh2a* embryos. n=1, 30 pooled embryos.

**Supplementary Figure 2: *rnaseh2a^-/-^* embryos are not highly sensitive to CPT treatment**
A) Treatment of *rnaseh2a^+/-^* incross with 500nM CPT from 4dpf to 5dpf revealed no significant difference in the genotypes of the most severely affected fish after blind selection. Scale Bar, 0.5mm B) Top: Treatment of *rnaseh2a^+/-^* incross with 10µM ATRi from 24hpf-3dpf revealed no difference in severity between genotypes. Bottom: Western blot and quantification of treated embryos shows reduction of pCHK1 in CPT and ATRi (Ceralasertib) treated embryos indicating drug activity. n=3 (20 pooled embryos), Unpaired t-test ±SD, *P*=0.350. C) Top: Treatment of *rnaseh2a^+/-^* incross with 10µM PARPi (Talazoparib) from 24hpf-3dpf revealed no difference in severity between genotypes. Bottom: Western blot and quantification of treated embryos shows reduction of PAR in treated embryos indicating drug activity. n=3 (20 pooled embryos), Unpaired t-test ±SD, *P*<0.0001 D) Top: Treatment of *rnaseh2a^+/-^* incross with 10µM Hydroxyurea from 24hpf-5dpf revealed no difference in severity between genotypes. Bottom: Western blot and quantification of treated embryos shows increased γH2AX indicating increased strand breakage, predicted after increased ribonucleotide incorporation. n=3 (20 pooled embryos), Unpaired t-test ±SD, *P*=0.1600. Representative images shown in all cases. E) Left: injection of 1.5nl of a pool of 2 guides targeting exon 1 and exon 2 of *rnaseh1* do not enhance *rnaseh2a* mutants, *rnaseh1* crispants appear slighty shorter than uninjected controls but there is no difference in response between *rnash2a* mutants and wild-type siblings. Right: PCR amplification of the target region showing ‘smudging” in injected specimens for each guide. This shows that both *rnaseh1* guides efficiently cause indels.
